# The association between liver fibrosis scores and chronic kidney disease

**DOI:** 10.3389/fmed.2023.1046825

**Published:** 2023-01-30

**Authors:** Shengjun Xiong, Pengbo Wang, Shizhang Yin, Wanshu Deng, Yuanhui Zhao, Wenhang Li, Zhao Li, Ying Zhou, Shasha Yu, Hongmei Yang, Xiaofan Guo, Yingxian Sun

**Affiliations:** Department of Cardiology, The First Hospital of China Medical University, Shenyang, Liaoning, China

**Keywords:** chronic kidney disease, liver fibrosis scores, BARD score, BAAT score, fibrosis-4

## Abstract

**Purpose:**

This study aimed to clarify the relationship between liver fibrosis scores (Fibrosis-4, BARD score, and BAAT score) and chronic kidney disease (CKD).

**Methods:**

We collected a range of data from 11,503 subjects (5,326 men and 6,177 women) from the rural regions of Northeastern China. Three liver fibrosis scores (LFSs) including fibrosis-4 (FIB-4), BARD score, and BAAT score were adopted. A logistic regression analysis was used to calculate odds ratios and the 95% confidence interval. A subgroup analysis showed the association between LFSs and CKD under different stratifications. Restricted cubic spline could further explore whether there is a linear relationship between LFSs and CKD. Finally, we used C-statistics, Net Reclassification Index (NRI), and Integrated Discrimination Improvement (IDI) to assess the effect of each LFS on CKD.

**Results:**

Through the baseline characteristics, we observed that LFSs were higher in the CKD population than in non-CKD. The proportion of participants with CKD also increased with LFSs. In a multivariate logistic regression analysis, the ORs of CKD were 6.71 (4.45–10.13) in FIB-4, 1.88 (1.29–2.75) in the BAAT score, and 1.72 (1.28–2.31) in the BARD score by comparing the high level with the low level in each LFSs. Moreover, after adding LFSs to the original risk prediction model, which consisted of age, sex, drinking, smoking, diabetes, low-density lipoprotein cholesterol, total cholesterol, triglycerides, and mean waist circumference, we found the new models have higher C-statistics. Furthermore, NRI and IDI both indicate LFSs had a positive effect on the model.

**Conclusions:**

Our study showed that LFSs are associated with CKD among middle-aged populations in rural areas of northeastern China.

## 1. Introduction

Chronic kidney disease (CKD) is becoming a heavy health burden in the world's adult population, affecting over 10–25% of people worldwide (1). CKD is defined as decreased estimated glomerular filtration (eGFR) (2). It is well known that cardiovascular events are a common complication of CKD. More than 50% of patients with CKD do not progress to the final stage due to high mortality from cardiovascular disease complications (3). Thus, we need to detect and prevent CKD in time.

It is believed that liver fibrosis is one of the common stages of chronic liver disease (4). Liver fibrosis is a determinant of clinically relevant events, including liver-related and cardio-metabolic events (5–8). Liver fibrosis score (LFS) is a non-invasive index used to evaluate liver fibrosis. LFS can play an important evaluation role when the patient is not a suitable candidate for liver biopsy (9). Some studies have suggested that liver fibrosis indicators may be associated with CKD (10, 11). However, the association between liver fibrosis scores and CKD in the general population has not been reported.

The liver fibrosis score is composed of traditional variables such as age, body mass index (BMI), and triglycerides. The commonly used LFSs are fibrosis-4 (FIB-4), the BAAT score, and the BARD score, which were included in our study. We organized this cross-sectional study in a general population to research the relationship between LFSs and CKD in the rural population of northeast China.

## 2. Methods

### 2.1. Study population

A large cross-sectional population study referred to as the Northeast China Rural Cardiovascular Health Study (NCRCHS) provided the data for our study. The NCRCHS used a multistage, stratified, random, and clustered sampling method. The design and principles of the NCRCHS have been described in detail elsewhere (12, 13). A representative group of adults were recruited from Liaoning Province as the research subject. Participants who were under 35 years old, pregnant, had mental health conditions, or had failed to complete the relevant assessments were excluded. Finally, 11,956 participants were included in our study. According to the criteria of the present study, we excluded 453 participants who had missing relevant information such as AST, ALT, hypertension, diabetes, BMI, drinking, smoking, and TG. In the end, we researched a target population of 11,503 for the present study. Our research was permitted by the Ethics Committee of China Medical University. After preliminary screening, the subjects provided their information and written consent to participate in our study. All data collection, storage, and analysis were performed in accordance with approved ethical protocols.

### 2.2. Data collection

Cardiologists collected data during a single clinical visit, and a standard questionnaire was used by trained nurses through face-to-face inquiry. Before conducting the survey, eligible investigators attended a training session and those who passed the training test were allowed to participate in our study. Our questionnaires obtained information on age, sex, and personal history. We also asked for details about the participants' lifestyles.

Data on smoking, drinking, education, family income, and other information were collected by us. Blood pressure was collected by two trained nurses using an automatic electronic sphygmomanometer (HEM-907; Omron, Kyoto, Japan). Blood pressures were measured three times and then the average was taken. We asked the subjects to take off their shoes before evaluating a range of anthropometric indices. The test results were accurate to 0.1 kg and 0.1 cm, respectively. All participants were told to fast for at least 12 h in advance before blood samples were collected the next morning. Blood samples were taken from the cubital veins to collect plasma levels of fasting glucose (FPG), triglycerides (TG), aspartate aminotransferase (AST), alanine aminotransferase (ALT), low-density lipoprotein cholesterol (LDL-C), and high-density lipoprotein cholesterol (HDL-C). A complete description of the procedures for storing blood samples and the measurement of laboratory indicators can be found elsewhere (14, 15).

### 2.3. Definitions

The Chronic Kidney Disease Epidemiology Collaboration (CKD-EPI) equation was used to calculate eGFR (16). The CKD-EPI equation is as follows: eGFR = 141 × min (Scr/κ, 1)α × max (Scr/κ, 1)−1.209 × 0.993Age × 1.018 [if woman], where Scr is serum creatinine, κ is 0.7 for women and 0.9 for men, α is −0.329 for women and −0.411 for men, min indicates the minimum of Scr/κ or 1, and max indicates the maximum of Scr/κ or 1. For practical purposes, CKD was defined as an eGFR of <60 mL/min per 1.73 m^2^ (17). CKD1 was defined as eGFR >90 mL/min per 1.73 m^2^; CKD2 was defined as eGFR 60–89 mL/min per 1.73 m^2^; CKD3 was defined as eGFR 30–59 mL/min per 1.73 m^2^; CKD4 was defined as eGFR >15–29 mL/min per 1.73 m^2^; CKD5 was defined as eGFR < 15 mL/min per 1.73 m^2^; and body mass index (BMI) was defined as body weight divided by height squared. Diabetes was defined as fasting blood sugar ≥7. The BARD score was calculated as BMI ≥ 28 kg/m^2^ (1 point) + AST/ALT ratio ≥ 0.8 (2 points) + presence of diabetes (1 point). The categories were: high (3–4 score, advanced fibrosis likely) or low (0–2 score, advanced fibrosis not likely) (18). APRI was calculated as AST/PLT× 100 (<0.5: low fibrosis, ≥0.5: high fibrosis) (19). BAAT score was calculated by the sum of the following: BMI ≥28 (1 point), age ≥50 years (1 point), male ALT ≥60 or female ALT ≥40 (1 point), and TG ≥ 1.7 mmol/L (1 point). The categories were: high (3–4 score, advanced fibrosis likely) or low (0–2 score, advanced fibrosis not likely) (20). FIB-4 scores were calculated using the following equation, with classification cutoffs of 1.30 and 2.67 (low:<1.30; intermediate:1.30–2.67; and high:>2.67): FIB-4 = age × AST/(platelet × ALT1/2) (21).

### 2.4. Data analysis

The values of continuous variables are expressed as the means ± standard deviations (SDs) or medians (Q1–Q3 quartiles) and numbers (percentages) for categorical variables. The differences in clinical characteristics between groups were analyzed using the Student's *t*-test, the Mann–Whitney U test, the analysis of variance (ANOVA), Fisher's exact test, or the chi-square test, where appropriate. We used multivariate logistic regression analyses to explore the independent association between LFSs and CKD. Univariate and adjusted odds ratios (ORs) and 95% confidence intervals (CI) were performed. These LFSs were analyzed as continuous variables (per 1 SD or per 1-point increment) and categorical variables with conventional cutoffs (as described above). The association between LFS and CKD was also examined by the restricted cubic spline (RCS) model. The predictive value added by LFSs in risk prediction models was assessed by C-statistic, continuous Net Reclassification Improvement (NRI), and Integrated Discrimination Improvement (IDI). For all analyses, two-tailed *P*-values of <0.05 were considered statistically significant. All statistical analyses were performed with SPSS version 26.0 software (SPSS Inc) and R version 4.1.1 (R Foundation for Statistical Computing).

## 3. Results

### 3.1. Baseline characteristics

There was a total of 11,503 subjects (5,326 men and 6,177 women) whose mean age was 53.9 ± 10.6 years. There were 243 participants with CKD and 36.6% were men. As shown in Table 1, age was the most obvious difference between CKD and non-CKD participants. Significantly, older age and lower eGFR were found in participants with CKD. The group with CKD had higher BMI, TG, TCH, LDL-c, and FPG. In the CKD group, the proportions of hypertension and diabetes were obviously higher than in the non-CKD group. All four LFSs in the CKD group were greatly higher than that in the non-CKD group. We also described the demographic and clinical characteristics of participants with different CKD stages, as shown in Supplementary Table S1. We found that ages were gradually increasing and liver fibrosis scores significantly increased as the stage of CKD progressed.

**Table 1:** Baseline characteristics of the study population.

**Variables**	**Total** **(*N =* 11,503)**	**CKD** **(*N =* 243)**	**Non-CKD** **(*N =* 11,260)**	***P*-value**
Age, year	53.9 ± 10.6	68.9 ± 9.4	53.6 ± 10.4	<0.001
Male sex, *n* (%)	5,326 (46.3)	89 (36.6)	5,237 (46.5)	<0.002
Body mass index, kg/m^2^	24.8 ± 3.7	25.0 ± 3.8	24.8 ± 3.7	0.430
Currently smoking, *n* (%)	4,045 (35.2)	69 (28.4)	3,976 (35.3)	0.019
Currently drinking, *n* (%)	2,576 (22.4)	16 (6.6)	2,560 (22.7)	<0.001
Hypertension, *n* (%)	5,816 (50.6)	191 (78.6)	5,625 (50.0)	<0.001
Diabetes, *n* (%)	1,201 (10.4)	62 (25.5)	1,139 (10.1)	<0.001
TG, mmol/L	1.6 ± 1.5	2.1 ± 1.7	1.6 ± 1.5	<0.001
TCH, mmol/L	5.2 ± 1.1	5.8 ± 1.6	5.2 ± 1.1	<0.001
LDL-C, mmol/L	2.9 ± 0.8	3.2 ± 1.1	2.9 ± 0.8	<0.001
HDL-C, mmol/L	1.4 ± 0.4	1.3 ± 0.4	1.4 ± 0.4	<0.001
ALT, IU/L	22.4 ± 18.2	18.4 ± 10.8	22.5 ± 18.3	<0.001
AST, IU/L	22.2 ± 11.9	20.4 ± 7.1	22.1 ± 11.9	0.025
PLT, 10/L	212.9 ± 66.7	207.3 ± 75.5	213.1 ± 66.5	0.24
eGFR, ml/min	93.9 ± 15.9	49.7 ± 11.5	93.9 ± 14.6	<0.001
FPG, mol/L	5.9 ± 1.6	6.6 ± 2.4	5.9 ± 1.6	<0.001
FIB-4	1.4 ± 1.0	1.9 ± 0.9	1.4 ± 1.0	<0.001
BAAT score	1.1 ± 0.9	1.7 ± 0.8	1.1 ± 0.9	<0.001
BARD score	1.9 ± 0.8	2.2 ± 0.8	1.9 ± 0.8	<0.001
APRI	0.29 ± 0.2	0.29 ± 0.2	0.28 ± 0.2	<0.001

Data are expressed as the mean value ± standard deviation or number (%). ALT, Alanine aminotransferase; AST, Aspartate aminotransferase; HDL-C, high-density lipoprotein cholesterol; LDL-C, low-density lipoprotein cholesterol; TCH, total cholesterol; TG, triglycerides; PLT, platelet; FPG, fasting plasma glucose; eGFR, estimated glomerular filtration rate.

### 3.2. Prevalence of CKD in different levels of FIB-4, APRI, BAAT, and BARD

**Figure 1:**
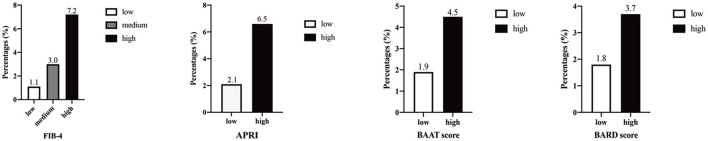
The prevalence of CKD by stratification of LFSs.

Figure 1 shows the prevalence of CKD at different stratification levels of LFSs. It was shown in FIB-4 (low: 1.1%, medium: 3.0%, and high: 7.2%), the APRI (low: 2.1 and high: 6.5%), the BAAT score (low: 1.9% and high: 4.5%), and the BARD score (low: 1.8% and high: 3.7%) that the prevalence of CKD progressively increased with LFSs. In addition, we described the prevalence of these CKD stages among participants with different levels of LFSs as shown in Supplementary Figure S1. We were able to see that the proportion of high-level liver fibrosis scores increased as the CKD stage progressed.

### 3.3. Univariate and adjusted odds ratio (95% CI) of FIB-4, APRI, BAAT, and BARD for CKD

To explore the association between CKD and LFSs, all subjects were analyzed using the logistic regression analysis, and the results are shown in Table 2. The odds ratios (ORs) for a 1 SD increase or 1 point increase in LFSs were FIB-4: 1.23 (1.14–1.33), APRI: 1.31 (1.05–1.54), BAAT score: 1.94 (1.70–2.21), and BARD score: 1.69 (1.43–2.00) in the univariate model. After adjustment for age, sex, smoking, drinking, hypertension, diabetes, LDL-C, TG, TCH, and mean waist circumference, the ORs were FIB-4: 1.21 (1.12–1.32), APRI: 1.18 (1.03–1.37), BAAT score: 1.56 (1.31–1.85), and BARD score: 1.34 (1.11–1.62). We further categorized the LFSs into different levels and compared the high level with the low level. The ORs of the high level were 6.86 times (FIB-4), 2.04 times (APRI), 2.41 times (BAAT score), and 2.13 times (BARD score) higher than those of the low level in the univariate model. After the same adjustments as above, the results were 6.71 times (FIB-4), 1.76 times (APRI), 1.88 times (BAAT score), and 1.72 times (BARD score).

**Table 2:** Univariate and multivariate logistic regression of LFSs for CKD.

**Variables**	**Total**	**Events (%)**	**Univariate Model**		**Adjusted Model**	
			**OR (95% CI)**	***P*-value**	**OR (95% CI)**	***P*-value**
**FIB-4**						
Per 1 SD increment			1.23 (1.14–1.33)	<0.001	1.21 (1.12–1.32)	<0.001
<1.3 (low)	6,639	74 (1.1)	1.00 (reference)		1.00 (reference)	
1.3–2.67 (medium)	4,307	129 (3.0)	2.74 (2.05–3.66)	<0.001	2.49 (1.86–3.34)	<0.001
>2.67 (high)	557	40 (7.2)	6.86 (4.62–10.19)	<0.001	6.71 (4.45–10.13)	<0.001
**APRI**						
Per 1 SD increment			1.31 (1.05–1.54)	<0.001	1.18 (1.03–1.37)	<0.001
≤0.5 (low)	10,748	227 (2.1)	1.00 (reference)		1.00 (reference)	
>0.5 (high)	755	49 (6.5)	2.04 (1.54–2.61)	<0.001	1.76 (1.32–2.23)	0.032
**BAAT score**						
Per 1 point increment			1.94 (1.70–2.21)	<0.001	1.56 (1.31–1.85)	<0.001
0–2 (low)	10,708	207 (1.9)	1.00 (reference)		1.00 (reference)	
3–4 (high)	795	36 (4.5)	2.41 (1.68–3.45)	<0.001	1.88 (1.29–2.75)	0.001
**BARD score**						
Per 1 point increment			1.69 (1.43–2.00)	<0.001	1.34 (1.11–1.62)	0.002
0–2 (low)	9,574	171 (1.8)	1.00 (reference)		1.00 (reference)	
3–4 (high)	1,929	72 (3.7)	2.13 (1.61–2.82)	<0.001	1.72 (1.28–2.31)	<0.001

Adjusted for age, sex, hypertension, smoking, drinking, diabetes, LDL-C, TG, TCH, mean waist circumference.

### 3.4. Restricted cubic spline (RCS) and subgroup analysis explored the relationship between LFSs and CKD

**Figure 2:**
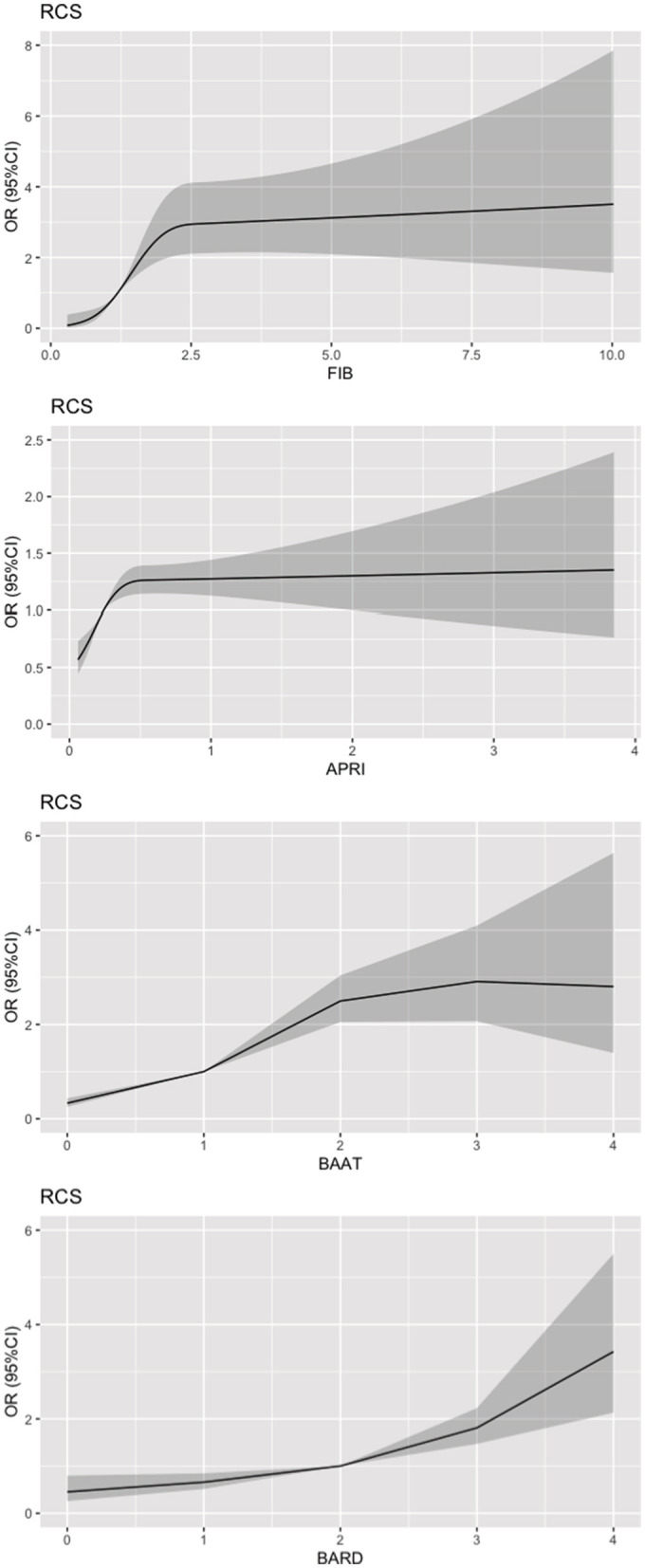
Restricted cubic spline plot of liver fibrosis scores and CKD. The vertical axis is the OR between LFS and CKD. The horizontal axis is the liver fibrosis score.

**Figure 3:**
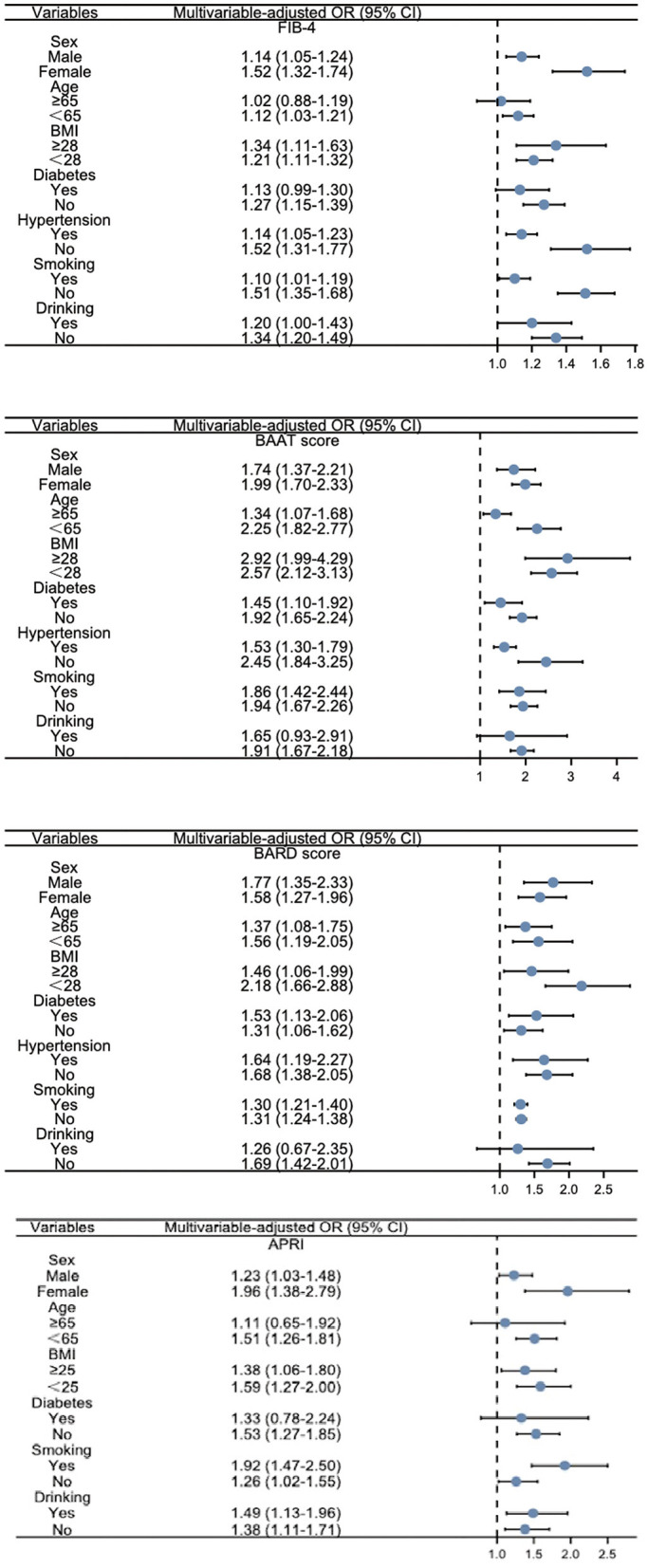
Subgroup analysis of LFSs in relation to CKD.

Restricted cubic spline was used to further investigate whether the association between LFSs and CKD was linear. As shown in Figure 2, only the BARD score showed a linear relationship, and the curves of FIB-4 and the BAAT score were not smooth and linear. Subgroup analysis was performed to explore the link between LFSs and CKD in different subgroups such as age, sex, diabetes, hypertension, BMI, smoking, and drinking. From Figure 3, we observed that the association between LFSs and CKD was still valid and significant in the majority of subgroups. After stratification by gender, the ORs for all three LFSs were statistically significant. In the FIB-4, the OR for men was 1.14 and for women, it was 1.52. In the APRI, the OR for men was 1.23 and for women, it was 1.96. In the BAAT score, the OR for men was 1.74 and for women, it was 1.99. In the BARD score, the OR for men was 1.77 and for women, it was 1.58. At the same time, since women's menstrual status has a great impact on their physical condition, we further stratified the group of women by whether they were menopausal or not. In menopausal women, the ORs for the three LFSs were 1.26 (FIB-4), 1.37 (APRI), 1.54 (BAAT score), and 1.49 (BARD score). In non-menopausal women, the ORs for the three LFSs were 2.57 (FIB-4), 1.64 (APRI), 1.79 (BAAT score), and 1.09 (BARD score).

### 3.5. C-statistic, NRI, and IDI explored the role of LFSs in the CKD risk model

In Table 3, the original model was made up of age, sex, drinking, smoking, diabetes, hypertension, LDL-C, TCH, TG, and mean waist circumference. The model was selected by finding the risk factors taken from relevant studies (22–24). We evaluated the calibration of the multivariate model by the Hosmer–Lemeshow goodness of fit test (*P* = 0.772), and the C-statistics of the original model was 0.739. After adding LFSs, respectively, to the original model, we found that the C-statistics of four new models were 0.767 (FIB-4), 0.765 (APRI), 0.762 (BAAT score), and 0.743 (BARD score). These values were all higher than the original model, which means LFSs have a positive effect on CKD. To accurately observe the role of LFS, we further performed the Net Reclassification Index (NRI) and the Integrated Discrimination Improvement (IDI). The NRI values of LFSs were all positive, which means LFSs have a positive contribution to the original model. The IDI values indicated that the improvement of LFSs for the model was 7.3% (FIB-4), 7.4% (APRI), 7.4% (BAAT score), and 7.3% (BARD score). The results of NRI and IDI further suggested that LFS had a positive effect on CKD.

**Table 3:** Discrimination and reclassification for CKD with the addition of LFSs to the traditional risk factors model.

**Model**	**C-statistics** **(95% CI)**	**NRI** **(95% CI)**	***P*-value**	**IDI** **(95% CI)**	***P*-value**
Original model	0.739 (0.709–0.769)				
Original model+FIB-4	0.767 (0.739–0.794)	1.029 (0.919–1.140)	<0.001	0.073 (0.059–0.087)	<0.001
Original model+APRI	0.765 (0.740–0.786)	1.020 (0.909–1.132)	<0.001	0.074 (0.061–0.089)	<0.001
Original model+BAAT	0.762 (0.735–0.789)	1.016 (0.906–1.126)	<0.001	0.074 (0.061–0.088)	<0.001
Original model+BARD	0.743 (0.712–0.773)	1.036 (0.926–1.145)	<0.001	0.073 (0.060–0.088)	<0.001

Original model: age, sex, drinking, smoking, hypertension, diabetes, LDL-C, TCH, TG, and mean waistline circumference.

## 4. Discussion

We explored the association between liver fibrosis scores and CKD in the total population and multiple subgroups. A significantly positive association between LFSs and CKD was demonstrated by our cross-sectional study that focused on a rural population in northeast China. Moreover, it was shown that adding these four LFSs to the original model, which included conventional risk factors, has a significant incremental impact on predictive value. This is the first study to explore the association between LFSs and CKD in rural China.

In our study, the proportion of patients with CKD increased with the level of LFSs, indicating that patients in the high-level group were at higher risk of CKD compared with those in the low-level group. In the baseline characteristics table, the average of four LFSs was higher in the CKD population, suggesting the same conclusion. In a multivariate logistic regression analysis, all four LFSs had significant ORs with CKD, even after adjusting for traditional risk factors. Additionally, after stratifying liver fibrosis scores, we found that the ORs of high-level LFSs were still statistically significant compared with low-level LFSs.

Growing evidence confirms that liver disease and chronic kidney disease are associated (25), providing the basis to explore the link between LFSs and CKD in the general population. It is well known that age is a common risk factor for CKD, and age is a component of FIB-4 and BAAT. This provides the basis for our speculation that LFSs are associated with CKD. The main risk factors for CKD are insulin resistance and hypercoagulability (high fibrinogen, factor VII, and von Willebrand factor levels) (2). The dietary habits of obese people can affect kidney function and structure, increasing the risk of CKD (26). Activation of the RAS system is thought to play a key role in the pathogenesis of obesity-related diseases, including CKD. It is a hallmark of obesity-related CKD that ACE–Ang II activation in the kidney leads to renal ectopic lipid deposition (27). Therefore, the fact that BAAT and BARD scores both contain BMI ≥ 28 may be a reason for their association with CKD. In addition, studies showed AST/ALT ratio is associated with insulin resistance, which may lead to the development of CKD, therefore, AST/ALT ratio has an effect on CKD (28, 29). LFSs contain AST or ALT, which gives evidence to our conclusion that the LFSs are associated with CKD. Therefore, liver fibrosis scores as a comprehensive scoring standard of risk factors for CKD should have a significant effect on CKD, which was consistent with the findings of our study. We stratified the risk of liver disease in the population by the level of the LFS score in the general population. When participants with high levels of LFS were found to be associated with CKD, we were able to infer that chronic liver disease would be associated with CKD.

Moreover, we performed restricted cubic spline (RCS) modeling to further investigate whether there was a linear relationship between LFSs and CKD. We observed that only the RCS of the BARD score showed a linear relationship. We consider that it may be because the components of the BARD score were all risk factors related to CKD such as BMI, AST/ALT, and diabetes, as previously stated. Then, we performed subgroup analysis and found that the ORs of different LFSs were variable in different stratification conditions, which can provide support for subsequent related studies under different population conditions. The gender differences we observed in our study might be attributed to a variety of factors such as lifestyle, diet, culture, and possibly the criteria of subjects. We also stratified by menopausal status and found that the association between LFS and CKD was more significant in menopausal women. In the menopausal state, the OR values were 1.26 in FIB-4, 1.54 in BAAT score, and 1.49 in BARD score. In the non-menopausal state, only the OR of FIB (2.57) was statistically significant. Studies have shown that women are more likely to be obese and have a higher BMI (30, 31). Furthermore, with the addition of the four LFSs, all the C-statistics increased, which indicated that the LFSs had a positive effect on predicting CKD. The Net Reclassification Index (NRI) and Integrated Discrimination Improvement (IDI) further demonstrated the usefulness of LFSs for the prediction of CKD. We consider our study to have clinical implications. The link between liver fibrosis and the kidney is increasingly being discovered, and our study also provides support for the link (32). For patients with liver fibrosis, we can prevent the development of CKD early on through LFSs.

Our study may have important clinical implications. We believe that liver fibrosis scores can help prevent CKD in the general population. Our findings strongly support the strategy of screening and monitoring CKD in the general population through liver fibrosis scores. Since these LFSs are well-validated measures, they can be easily calculated using routine clinical laboratory results. We can use them to assist clinicians with kidney disease counseling and monitoring to reduce the risk of kidney disease in the general population. They can serve as valuable tools for large population-based epidemiological studies. In addition, there is growing evidence that liver fibrosis increased insulin resistance, which increases free fatty acids and causes systemic vasculitis. It would lead to dyslipidemia and releases a variety of pro-inflammatory and pro-fibrotic molecules that can contribute to the development of CKD (33). In addition, the progression of liver fibrosis is hemodynamically considered to be a progression of chronic inflammation (2). The inflammatory cytokines caused by liver fibrosis also act on the kidney, causing chronic inflammation in the renal vasculature as well (34). Our findings provided new perspectives on renal–liver multisystem diseases and suggested the need for more basic research on the mechanisms of liver fibrosis as a driver of incident CKD.

Through the results of the study, we have an interesting finding that FIB-4 might be the most superior of the three LFSs in correlation with CKD. In logistic regression, the OR of high-level FIB-4 was the highest (6.71), and the C-statistic of the risk model after adding the FIB is also the highest (0.767). The reason for this finding could be explained by the variables included in the scores. FIB-4 score includes factors associated with the development of fibrosis, such as age, and factors associated with cirrhosis, such as platelets (35). Furthermore, FIB-4 included AST/platelet ratio and AST/ALT1/2 which are good indicators of more advanced fibrosis and cirrhosis in liver disease (36). Both BAAT and BARD are simple scores and do not include variables for the development of chronic liver disease. Therefore, FIB-4 might be the preferred score.

This study has some limitations. First, with the study having a cross-sectional design, only the association between liver fibrosis scores and CKD was determined. The timeline is not taken into account by our analysis. Second, our participants were from rural northeastern China, and these results may not apply to populations from other regions or ethnicities. Since the diagnosis of liver disease requires a variety of biochemical indicators as well as medical imaging tests, we did not have sufficient conditions to diagnose liver disease in our participants. We could not completely exclude the presence of unrecognized liver disease in the participants, which would cause bias in the results of the study. Finally, we do not have therapy information for the population, which may lead to a certain bias in our results.

In conclusion, our study found a clear association between LFSs and CKD and a positive effect of LFSs in predicting the risk of CKD.

## Data availability statement

The original contributions presented in the study are included in the article/Supplementary material, further inquiries can be directed to the corresponding author.

## Ethics statement

The studies involving human participants were reviewed and approved by the Ethics Committee of China Medical University (Shenyang, China; ethical approved project identification code: AF-SOP-07-1, 0-01). The patients/participants provided their written informed consent to participate in this study.

## Author contributions

SX, HY, XG, and YS contributed to conception and design of the study. PW and ZL organized the database. YZho and YS performed the statistical analysis. SX wrote the first draft of the manuscript. SYin, WD, YZha, and WL performed data curation. All authors contributed to manuscript revision, read, and approved the submitted version. All authors have given their consent for publication of this article.
