# Supplementary figures and images for: The association between liver fibrosis scores and chronic kidney disease
Source: Front Med (Lausanne). 2023 Jan 30;10:1046825. doi: 10.3389/fmed.2023.1046825 (PMC9922852; doi:10.3389/fmed.2023.1046825)

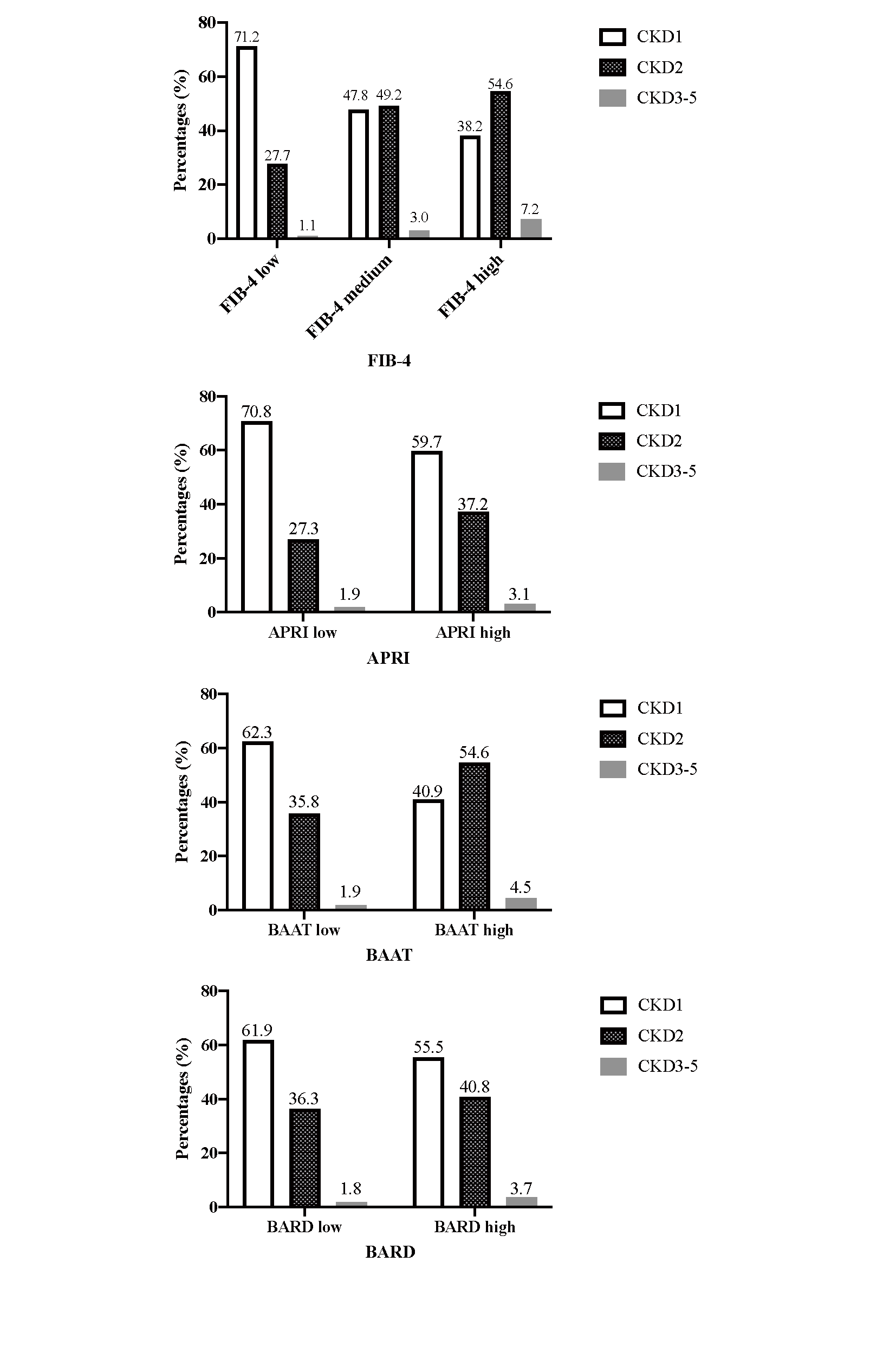

Supplement: Supplementary file 1 [file Image_1.tiff]
