# Supplementary material for: The association between liver fibrosis scores and chronic kidney disease
Source: Front Med (Lausanne). 2023 Jan 30;10:1046825. doi: 10.3389/fmed.2023.1046825 (PMC9922852; doi:10.3389/fmed.2023.1046825)
Supplement: Supplementary file 2 [file Table_1.docx]

Table S1. Baseline characteristics of the study population with different CKD stages.

| Variables | CKD1  (N=6995) | CKD2  (N=4265) | CKD3  (N=225) | CKD4  (N=10) | CKD5  (N=8) | P value |
| --- | --- | --- | --- | --- | --- | --- |
| Age, year | 49.9±8.7 | 59.6±10.1 | 69.5±9.1 | 69.9±10.8 | 72.4±9.3 | ＜0.001 |
| Male sex, n (%) | 3509 (50.2) | 1728 (40.5) | 82 (36.5) | 4 (40) | 3 (37.5) | 0.372 |
| Body mass index, kg/m2 | 24.8±3.7 | 24.8±3.6 | 30.0±3.9 | 26.6±3.6 | 23.6±1.6 | 0.531 |
| Currently smoking, n (%) | 2655 (38) | 1321 (31) | 65 (28.9) | 3 (30) | 5 (62.5) | 0.009 |
| Currently drinking, n (%) | 1882 (26.9) | 678 (15.9) | 14 (6.2) | 2 (20) | 8 (100) | 0.032 |
| Hypertension, n (%) | 3142 (44.9) | 2483 (58.2) | 173 (76.9) | 10 (100) | 8 (100) | 0.024 |
| Diabetes, n (%) | 613 (8.8) | 526 (12.3) | 57 (25.3) | 5 (50) | 6 (75) | 0.003 |
| TG, mmol/L | 1.6±1.5 | 1.7±1.3 | 2.1±1.7 | 2.5±1.4 | 2.4±0.3 | 0.31 |
| TCH, mmol/L | 5.1±1.0 | 5.4±1.4 | 5.8±1.7 | 5.5±1.5 | 5.9±1.2 | 0.05 |
| LDL-C, mmol/L | 2.9±0.8 | 3.0±0.8 | 3.3±1.1 | 3.1±0.9 | 23.8±0.8 | ＜0.001 |
| HDL-C, mmol/L | 1.4±0.4 | 1.3±0.4 | 1.4±0.4 | 1.1±0.2 | 1.3±0.2 | 0.14 |
| ALT, IU/L | 23.1±20.1 | 21.5±15.0 | 22.5±18.3 | 23.8±26.3 | 14.7±8.1 | 0.33 |
| AST, IU/L | 22.6±13.2 | 21.4±9.4 | 22.1±11.9 | 22.8±8.6 | 14.4±3.9 | 0.37 |
| PLT, 10/L | 218.6±70.5 | 204.0±58.2 | 204.7±66.1 | 230.6±68.8 | 214.8±90.5 | 0.22 |
| FPG, mol/L | 5.8±1.7 | 6.0±1.5 | 6.6±2.4 | 7.1±2.3 | 5.5±0.6 | ＜0.001 |
| FIB-4 | 1.2±1.0 | 1.5±0.9 | 1.9±0.9 | 2.0±0.9 | 2.2±0.7 | ＜0.001 |
| APRI | 0.3±0.3 | 0.3±0.2 | 0.3±0.3 | 0.3±0.1 | 0.3±0.1 | ＜0.001 |
| BAAT score | 0.9±0.9 | 1.4±0.8 | 1.7±0.9 | 2.1±1.0 | 2.8±0.6 | ＜0.001 |
| BARD score | 1.8±0.8 | 1.9±0.9 | 2.2±0.8 | 2.7±0.7 | 2.5±0.9 | ＜0.001 |

Data are expressed as the mean value ± standard deviation or number (%). Abbreviations: ALT, Alanine aminotransferase; AST, Aspartate aminotransferase; HDL-C, high-density lipoprotein cholesterol; LDL-C, low-density lipoprotein cholesterol; TCH, total cholesterol; TG, triglycerides; PLT, platelet;
